# Crystal structure of (*E*)-4,6-dimeth­oxy-2-(4-meth­oxy­styr­yl)-3-methyl­benzaldehyde

**DOI:** 10.1107/S2056989015017363

**Published:** 2015-09-26

**Authors:** Seunghyun Ahn, Yoongho Lim, Dongsoo Koh

**Affiliations:** a Division of Bioscience and Biotechnology, BMIC, Konkuk University, Seoul 143-701, Republic of Korea; b Department of Applied Chemistry, Dongduk Women’s University, Seoul 136-714, Republic of Korea

**Keywords:** crystal structure, benzaldehyde, resveratrol derivatives, biological properties

## Abstract

In the title mol­ecule, C_19_H_20_O_4_, the central C=C double bond adopts an *E* configuration. The dihedral angle formed by the planes of the two benzene rings is 83.57 (12)°. The three meth­oxy groups are essentially coplanar with the benzene rings to which they are attached, with C C—O—C torsion angles of −0.2 (3), −2.3 (3) and −4.1 (3)°.

## Related literature   

For the synthesis and biological properties of resveratrol derivatives, see: Chen *et al.* (2015 ▸); Chillemi *et al.* (2015 ▸); Li *et al.* (2014 ▸); Shin *et al.* (2014 ▸); Huang *et al.* (2007 ▸). For related structures, see: Ge *et al.* (2013 ▸); Tang *et al.* (2011 ▸).
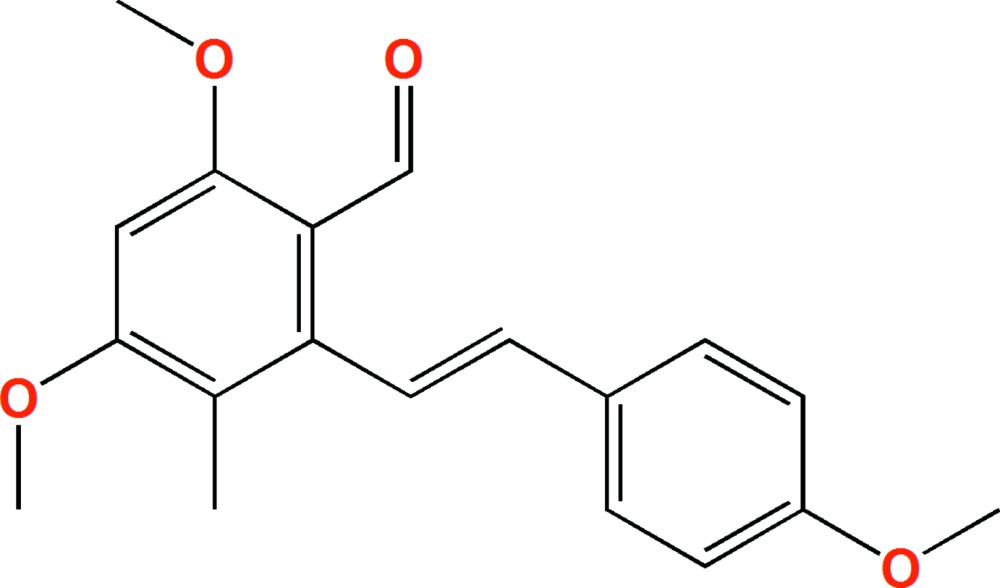


## Experimental   

### Crystal data  

C_19_H_20_O_4_
*M* *_r_* = 312.35
Monoclinic, 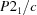

*a* = 11.3632 (9) Å
*b* = 8.7159 (7) Å
*c* = 16.2382 (13) Å
β = 101.927 (2)°
*V* = 1573.5 (2) Å^3^
*Z* = 4
Mo *K*α radiation
μ = 0.09 mm^−1^
*T* = 200 K
0.26 × 0.20 × 0.13 mm

### Data collection  

Bruker SMART CCD diffractometer
11266 measured reflections
3904 independent reflections
1966 reflections with *I* > 2σ(*I*)
*R*_int_ = 0.037

### Refinement  

*R*[*F* ^2^ > 2σ(*F* ^2^)] = 0.057
*wR*(*F* ^2^) = 0.222
*S* = 0.99
3904 reflections
212 parameters
H-atom parameters constrained
Δρ_max_ = 0.24 e Å^−3^
Δρ_min_ = −0.32 e Å^−3^

### 

Data collection: *SMART* (Bruker, 2000 ▸); cell refinement: *SAINT* (Bruker, 2000 ▸); data reduction: *SAINT*; program(s) used to solve structure: *SHELXS97* (Sheldrick, 2008 ▸); program(s) used to refine structure: *SHELXL97* (Sheldrick, 2008 ▸); molecular graphics: *SHELXTL* (Sheldrick, 2008 ▸); software used to prepare material for publication: *SHELXTL*.

## Supplementary Material

Crystal structure: contains datablock(s) I, New_Global_Publ_Block. DOI: 10.1107/S2056989015017363/lh5786sup1.cif

Structure factors: contains datablock(s) I. DOI: 10.1107/S2056989015017363/lh5786Isup2.hkl

Supporting information file. DOI: 10.1107/S2056989015017363/lh5786Isup3.cml

**Fig. 1.**
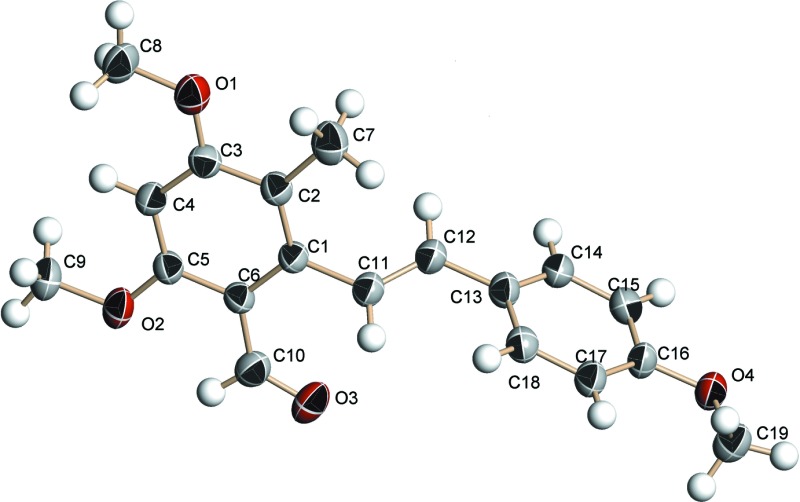
The molecular structure of the title compound, showing the atom labelling scheme and displacement ellipsoids drawn at the 30% probability level.

. DOI: 10.1107/S2056989015017363/lh5786fig1.tif
The mol­ecular structure of the title compound, showing the atom labelling scheme and displacement ellipsoids drawn at the 30% probability level.

**Fig. 2.**
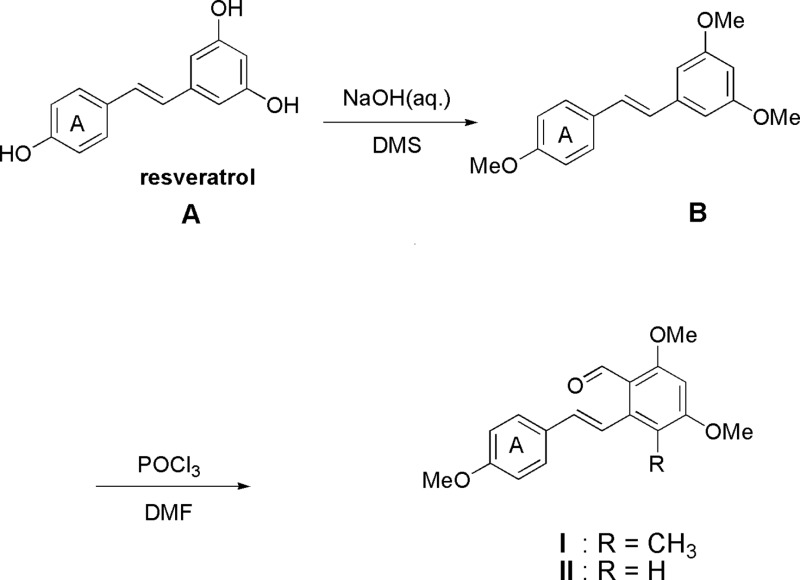
Synthetic scheme for preparation of resveratrol derivative compounds.

. DOI: 10.1107/S2056989015017363/lh5786fig2.tif
Synthetic scheme for preparation of resveratrol derivative compounds.

CCDC reference: 1425329

Additional supporting information:  crystallographic information; 3D view; checkCIF report

## supplementary crystallographic information

### S1. Introduction

Resveratrol is part of a family of the stilbene polyphenols which has a general C6—C2—C6 carbon framework. Recent research has shown that resveratrol derivatives have diverse biological activities including anti-Alzheimer's disease (Li *et al.*, 2014), anti­cancer (Chillemi *et al.*, 2015), and anti-inflammatory (Chen *et al.*, 2015). On our going research project of polyphenols (Shin *et al.*, 2014), the title compound was synthesized and its crystal structure was determined.

After the methyl­ation of hydroxyl groups in resveratrol, formyl­ation of the resulting compound was performed (Fig 2). The Vilslmeier formyl­ation reaction gave two products depending on reaction conditions (Huang *et al.*, 2007). The title compound (I) contains one formyl group and one methyl group at each ortho position of the di­meth­oxy-substituted benzene ring. Whereas compound (II) has only a formyl group at the ortho position of the benzene ring. The crystal structure of compound (II) has been published recently (Ge *et al.* 2013). In this report, the title compound (I) was synthesized and its crystal structure was determined. According to the literature (Ge *et al.* 2013), compound (II) contains two independent molecules in the asymmetric unit and the dihedral angle between the two benzene rings in each are 23.54 (12)°, and 31.11 (12)°. However, in (I), dihedral angle between the two benzene rings is 83.57 (12)° (Fig. 1). The meth­oxy groups are essentially co-planar with the attached the benzene rings [C4—C3—O1—C8 = -0.2 (3)°, C4—C5—O2—C9 = -2.3 (3)° and C17—C16—O4—C19 = -4.1 (3)°].

### S2. Experimental

For an outline of the synthesis see Fig. 2. Resveratrol (A, 30 mmol, 6.8 g) was dissolved in 75 mL of aq. NaOH (10%) under ice-water bath conditions. To the above solution, was added DMS (50 mL) and the reaction mixture was stirred at room temperature for 24 h. After completion of this reaction, the mixture was extracted with EtOAc (50 mL x 3) and the combined organic layer was dried under MgSO_4_. Filtration and evaporation of the solvent gave a solid of compound B. To a solution of compound B (10 mmol, 2.7 g) in 20 mL of DMF was added 2 mL of POCl_3_ in an ice-water bath, and was stirred at room temperature for 4 h. The reaction mixture was poured into ice-water and stirred for 2 h. The reaction mixture was extracted with EtOAc (30 mL x 3). Evaporation of the organic solvent afforded mixture of products (I) and (II), which was purified by column chromatography. Recrystallization of solid (I) from ethanol gave single crystals which were suitable for X-ray diffraction (m.p.: 391–392 K).

### S2.1. Refinement

H atoms bonded to C atoms were placed in calculated positions, with C—H distances in the range 0.95–0.98 Å, and included in the refinement in a riding-model approximation, with *U*_iso_(H) = 1.5*U*_eq_(C) for methyl H atoms and 1.5*U*_eq_(C) otherwise.

### Crystal data

**Table:**

C_19_H_20_O_4_	*F*(000) = 664
*M**_r_* = 312.35	*D*_x_ = 1.318 Mg m^−^^3^
Monoclinic, *P*2_1_/*c*	Mo *K*α radiation, λ = 0.71073 Å
Hall symbol: -P 2ybc	Cell parameters from 3256 reflections
*a* = 11.3632 (9) Å	θ = 2.7–28.2°
*b* = 8.7159 (7) Å	µ = 0.09 mm^−^^1^
*c* = 16.2382 (13) Å	*T* = 200 K
β = 101.927 (2)°	Block, light yellow
*V* = 1573.5 (2) Å^3^	0.26 × 0.20 × 0.13 mm
*Z* = 4	

### Data collection

**Table:**

Bruker SMART CCD diffractometer	1966 reflections with *I* > 2σ(*I*)
Radiation source: fine-focus sealed tube	*R*_int_ = 0.037
Graphite monochromator	θ_max_ = 28.3°, θ_min_ = 1.8°
φ and ω scans	*h* = −15→14
11266 measured reflections	*k* = −11→11
3904 independent reflections	*l* = −16→21

### Refinement

**Table:**

Refinement on *F*^2^	Primary atom site location: structure-invariant direct methods
Least-squares matrix: full	Secondary atom site location: difference Fourier map
*R*[*F*^2^ > 2σ(*F*^2^)] = 0.057	Hydrogen site location: inferred from neighbouring sites
*wR*(*F*^2^) = 0.222	H-atom parameters constrained
*S* = 0.99	*w* = 1/[σ^2^(*F*_o_^2^) + (0.1179*P*)^2^] where *P* = (*F*_o_^2^ + 2*F*_c_^2^)/3
3904 reflections	(Δ/σ)_max_ < 0.001
212 parameters	Δρ_max_ = 0.24 e Å^−^^3^
0 restraints	Δρ_min_ = −0.31 e Å^−^^3^

### Special details

**Table:**

Geometry. All e.s.d.'s (except the e.s.d. in the dihedral angle between two l.s. planes) are estimated using the full covariance matrix. The cell e.s.d.'s are taken into account individually in the estimation of e.s.d.'s in distances, angles and torsion angles; correlations between e.s.d.'s in cell parameters are only used when they are defined by crystal symmetry. An approximate (isotropic) treatment of cell e.s.d.'s is used for estimating e.s.d.'s involving l.s. planes.
Refinement. Refinement of *F*^2^ against ALL reflections. The weighted *R*-factor *wR* and goodness of fit *S* are based on *F*^2^, conventional *R*-factors *R* are based on *F*, with *F* set to zero for negative *F*^2^. The threshold expression of *F*^2^ > σ(*F*^2^) is used only for calculating *R*-factors(gt) *etc*. and is not relevant to the choice of reflections for refinement. *R*-factors based on *F*^2^ are statistically about twice as large as those based on *F*, and *R*- factors based on ALL data will be even larger.

### Fractional atomic coordinates and isotropic or equivalent isotropic displacement parameters (Å^2^)

**Table:**

	*x*	*y*	*z*	*U*_iso_*/*U*_eq_	
C1	0.8260 (2)	0.0967 (3)	0.05627 (14)	0.0374 (5)	
C2	0.7634 (2)	0.0081 (3)	−0.01021 (15)	0.0400 (6)	
C3	0.7955 (2)	0.0212 (3)	−0.08861 (14)	0.0398 (6)	
C4	0.8854 (2)	0.1179 (3)	−0.10269 (15)	0.0408 (6)	
H4	0.9040	0.1253	−0.1569	0.049*	
C5	0.9481 (2)	0.2042 (3)	−0.03599 (14)	0.0384 (6)	
C6	0.9196 (2)	0.1962 (2)	0.04419 (14)	0.0368 (5)	
C7	0.6658 (2)	−0.1046 (3)	−0.00234 (18)	0.0568 (7)	
H7A	0.5871	−0.0586	−0.0248	0.085*	
H7B	0.6718	−0.1308	0.0571	0.085*	
H7C	0.6752	−0.1978	−0.0342	0.085*	
O1	0.73156 (16)	−0.0693 (2)	−0.15023 (11)	0.0533 (5)	
C8	0.7593 (3)	−0.0620 (3)	−0.23169 (15)	0.0582 (8)	
H8A	0.7488	0.0435	−0.2529	0.087*	
H8B	0.7054	−0.1304	−0.2700	0.087*	
H8C	0.8429	−0.0940	−0.2283	0.087*	
O2	1.03780 (16)	0.30232 (19)	−0.04472 (10)	0.0481 (5)	
C9	1.0693 (2)	0.3103 (3)	−0.12493 (16)	0.0516 (7)	
H9A	1.0958	0.2090	−0.1400	0.077*	
H9B	1.1346	0.3846	−0.1229	0.077*	
H9C	0.9991	0.3428	−0.1672	0.077*	
O3	0.97338 (18)	0.3109 (2)	0.18130 (11)	0.0574 (5)	
C10	0.9879 (2)	0.2931 (3)	0.10982 (16)	0.0477 (6)	
H10	1.0519	0.3497	0.0950	0.057*	
C11	0.7965 (2)	0.0880 (3)	0.14099 (14)	0.0404 (6)	
H11	0.8607	0.0662	0.1871	0.049*	
C12	0.6884 (2)	0.1081 (3)	0.15791 (15)	0.0417 (6)	
H12	0.6232	0.1226	0.1114	0.050*	
C13	0.6605 (2)	0.1098 (3)	0.24196 (15)	0.0414 (6)	
C14	0.5693 (2)	0.2034 (3)	0.25915 (15)	0.0464 (6)	
H14	0.5208	0.2594	0.2145	0.056*	
C15	0.5475 (2)	0.2171 (3)	0.33950 (16)	0.0483 (6)	
H15	0.4861	0.2839	0.3498	0.058*	
C16	0.6156 (2)	0.1327 (3)	0.40527 (14)	0.0405 (6)	
C17	0.7041 (2)	0.0355 (3)	0.38910 (15)	0.0441 (6)	
H17	0.7495	−0.0247	0.4332	0.053*	
C18	0.7270 (2)	0.0256 (3)	0.30880 (14)	0.0437 (6)	
H18	0.7894	−0.0400	0.2989	0.052*	
O4	0.58605 (15)	0.15293 (19)	0.48210 (10)	0.0480 (5)	
C19	0.6468 (2)	0.0587 (3)	0.54938 (15)	0.0524 (7)	
H19A	0.6389	−0.0492	0.5322	0.079*	
H19B	0.6112	0.0739	0.5988	0.079*	
H19C	0.7322	0.0867	0.5633	0.079*	

### Atomic displacement parameters (Å^2^)

**Table:**

	*U*^11^	*U*^22^	*U*^33^	*U*^12^	*U*^13^	*U*^23^
C1	0.0382 (12)	0.0403 (12)	0.0348 (12)	0.0087 (10)	0.0102 (10)	0.0032 (10)
C2	0.0396 (13)	0.0408 (13)	0.0406 (13)	0.0040 (10)	0.0110 (10)	−0.0006 (10)
C3	0.0390 (13)	0.0406 (12)	0.0399 (13)	0.0023 (10)	0.0080 (10)	−0.0043 (11)
C4	0.0433 (13)	0.0454 (13)	0.0348 (12)	0.0052 (11)	0.0109 (10)	0.0012 (10)
C5	0.0375 (12)	0.0407 (12)	0.0389 (13)	0.0049 (10)	0.0124 (10)	0.0022 (10)
C6	0.0344 (12)	0.0388 (12)	0.0381 (12)	0.0048 (10)	0.0094 (10)	0.0046 (10)
C7	0.0569 (17)	0.0580 (16)	0.0592 (18)	−0.0107 (13)	0.0205 (14)	−0.0078 (14)
O1	0.0529 (11)	0.0631 (11)	0.0452 (10)	−0.0100 (9)	0.0130 (8)	−0.0147 (9)
C8	0.0631 (18)	0.0748 (19)	0.0369 (14)	−0.0077 (15)	0.0108 (12)	−0.0130 (13)
O2	0.0517 (10)	0.0557 (11)	0.0409 (10)	−0.0137 (8)	0.0193 (8)	−0.0016 (8)
C9	0.0583 (16)	0.0561 (15)	0.0462 (15)	−0.0048 (13)	0.0246 (13)	0.0014 (12)
O3	0.0725 (13)	0.0651 (12)	0.0353 (10)	−0.0099 (9)	0.0130 (9)	−0.0052 (9)
C10	0.0480 (15)	0.0541 (15)	0.0405 (14)	−0.0051 (12)	0.0077 (12)	−0.0004 (12)
C11	0.0437 (13)	0.0422 (12)	0.0370 (13)	0.0014 (10)	0.0121 (10)	0.0023 (10)
C12	0.0430 (14)	0.0477 (14)	0.0360 (12)	−0.0019 (11)	0.0116 (10)	0.0014 (11)
C13	0.0404 (13)	0.0461 (13)	0.0398 (13)	−0.0044 (10)	0.0136 (10)	0.0006 (11)
C14	0.0417 (14)	0.0613 (16)	0.0375 (13)	0.0047 (11)	0.0109 (11)	0.0046 (12)
C15	0.0411 (14)	0.0581 (15)	0.0479 (15)	0.0023 (12)	0.0146 (11)	−0.0002 (12)
C16	0.0406 (13)	0.0494 (13)	0.0339 (12)	−0.0095 (11)	0.0129 (10)	−0.0052 (11)
C17	0.0459 (14)	0.0482 (14)	0.0410 (13)	0.0013 (11)	0.0150 (11)	0.0058 (11)
C18	0.0432 (13)	0.0451 (13)	0.0454 (14)	0.0024 (11)	0.0150 (11)	0.0022 (11)
O4	0.0497 (10)	0.0588 (11)	0.0382 (9)	−0.0032 (8)	0.0154 (8)	−0.0025 (8)
C19	0.0594 (16)	0.0543 (15)	0.0431 (14)	−0.0127 (13)	0.0100 (12)	0.0003 (12)

### Geometric parameters (Å, º)

**Table:**

C1—C2	1.396 (3)	C9—H9C	0.9800
C1—C6	1.417 (3)	O3—C10	1.215 (3)
C1—C11	1.484 (3)	C10—H10	0.9500
C2—C3	1.399 (3)	C11—C12	1.325 (3)
C2—C7	1.506 (3)	C11—H11	0.9500
C3—O1	1.360 (3)	C12—C13	1.463 (3)
C3—C4	1.379 (3)	C12—H12	0.9500
C4—C5	1.388 (3)	C13—C14	1.391 (3)
C4—H4	0.9500	C13—C18	1.396 (3)
C5—O2	1.360 (3)	C14—C15	1.383 (3)
C5—C6	1.407 (3)	C14—H14	0.9500
C6—C10	1.453 (3)	C15—C16	1.393 (3)
C7—H7A	0.9800	C15—H15	0.9500
C7—H7B	0.9800	C16—O4	1.369 (3)
C7—H7C	0.9800	C16—C17	1.381 (3)
O1—C8	1.423 (3)	C17—C18	1.384 (3)
C8—H8A	0.9800	C17—H17	0.9500
C8—H8B	0.9800	C18—H18	0.9500
C8—H8C	0.9800	O4—C19	1.426 (3)
O2—C9	1.422 (3)	C19—H19A	0.9800
C9—H9A	0.9800	C19—H19B	0.9800
C9—H9B	0.9800	C19—H19C	0.9800
			
C2—C1—C6	120.5 (2)	H9A—C9—H9C	109.5
C2—C1—C11	120.8 (2)	H9B—C9—H9C	109.5
C6—C1—C11	118.6 (2)	O3—C10—C6	128.1 (2)
C1—C2—C3	118.1 (2)	O3—C10—H10	115.9
C1—C2—C7	124.1 (2)	C6—C10—H10	115.9
C3—C2—C7	117.7 (2)	C12—C11—C1	125.6 (2)
O1—C3—C4	122.2 (2)	C12—C11—H11	117.2
O1—C3—C2	115.0 (2)	C1—C11—H11	117.2
C4—C3—C2	122.8 (2)	C11—C12—C13	125.7 (2)
C3—C4—C5	118.7 (2)	C11—C12—H12	117.1
C3—C4—H4	120.7	C13—C12—H12	117.1
C5—C4—H4	120.7	C14—C13—C18	117.3 (2)
O2—C5—C4	122.3 (2)	C14—C13—C12	120.4 (2)
O2—C5—C6	116.7 (2)	C18—C13—C12	122.2 (2)
C4—C5—C6	121.1 (2)	C15—C14—C13	121.7 (2)
C5—C6—C1	118.8 (2)	C15—C14—H14	119.1
C5—C6—C10	117.4 (2)	C13—C14—H14	119.1
C1—C6—C10	123.9 (2)	C14—C15—C16	119.9 (2)
C2—C7—H7A	109.5	C14—C15—H15	120.1
C2—C7—H7B	109.5	C16—C15—H15	120.1
H7A—C7—H7B	109.5	O4—C16—C17	125.3 (2)
C2—C7—H7C	109.5	O4—C16—C15	115.4 (2)
H7A—C7—H7C	109.5	C17—C16—C15	119.3 (2)
H7B—C7—H7C	109.5	C16—C17—C18	120.2 (2)
C3—O1—C8	118.1 (2)	C16—C17—H17	119.9
O1—C8—H8A	109.5	C18—C17—H17	119.9
O1—C8—H8B	109.5	C17—C18—C13	121.5 (2)
H8A—C8—H8B	109.5	C17—C18—H18	119.2
O1—C8—H8C	109.5	C13—C18—H18	119.2
H8A—C8—H8C	109.5	C16—O4—C19	116.92 (19)
H8B—C8—H8C	109.5	O4—C19—H19A	109.5
C5—O2—C9	117.52 (18)	O4—C19—H19B	109.5
O2—C9—H9A	109.5	H19A—C19—H19B	109.5
O2—C9—H9B	109.5	O4—C19—H19C	109.5
H9A—C9—H9B	109.5	H19A—C19—H19C	109.5
O2—C9—H9C	109.5	H19B—C19—H19C	109.5
			
C6—C1—C2—C3	−0.3 (3)	C4—C5—O2—C9	−2.2 (3)
C11—C1—C2—C3	−179.9 (2)	C6—C5—O2—C9	179.0 (2)
C6—C1—C2—C7	177.8 (2)	C5—C6—C10—O3	175.2 (2)
C11—C1—C2—C7	−1.9 (3)	C1—C6—C10—O3	−4.0 (4)
C1—C2—C3—O1	179.49 (19)	C2—C1—C11—C12	−53.9 (3)
C7—C2—C3—O1	1.3 (3)	C6—C1—C11—C12	126.4 (3)
C1—C2—C3—C4	−0.3 (3)	C1—C11—C12—C13	−175.6 (2)
C7—C2—C3—C4	−178.5 (2)	C11—C12—C13—C14	146.5 (3)
O1—C3—C4—C5	−178.7 (2)	C11—C12—C13—C18	−29.9 (4)
C2—C3—C4—C5	1.0 (3)	C18—C13—C14—C15	2.1 (4)
C3—C4—C5—O2	−179.9 (2)	C12—C13—C14—C15	−174.6 (2)
C3—C4—C5—C6	−1.2 (3)	C13—C14—C15—C16	−1.7 (4)
O2—C5—C6—C1	179.4 (2)	C14—C15—C16—O4	−179.2 (2)
C4—C5—C6—C1	0.7 (3)	C14—C15—C16—C17	−0.3 (4)
O2—C5—C6—C10	0.2 (3)	O4—C16—C17—C18	−179.3 (2)
C4—C5—C6—C10	−178.6 (2)	C15—C16—C17—C18	1.8 (4)
C2—C1—C6—C5	0.1 (3)	C16—C17—C18—C13	−1.4 (4)
C11—C1—C6—C5	179.75 (19)	C14—C13—C18—C17	−0.5 (3)
C2—C1—C6—C10	179.3 (2)	C12—C13—C18—C17	176.1 (2)
C11—C1—C6—C10	−1.0 (3)	C17—C16—O4—C19	−4.1 (3)
C4—C3—O1—C8	−0.2 (3)	C15—C16—O4—C19	174.8 (2)
C2—C3—O1—C8	−180.0 (2)		
