# Supplementary figures and images for: Crystal structure of (E)-4,6-dimeth­oxy-2-(4-meth­oxy­styr­yl)-3-methyl­benzaldehyde
Source: Acta Crystallogr E Crystallogr Commun. 2015 Sep 26;71(Pt 10):o771. doi: 10.1107/S2056989015017363 (PMC4647390; doi:10.1107/S2056989015017363)

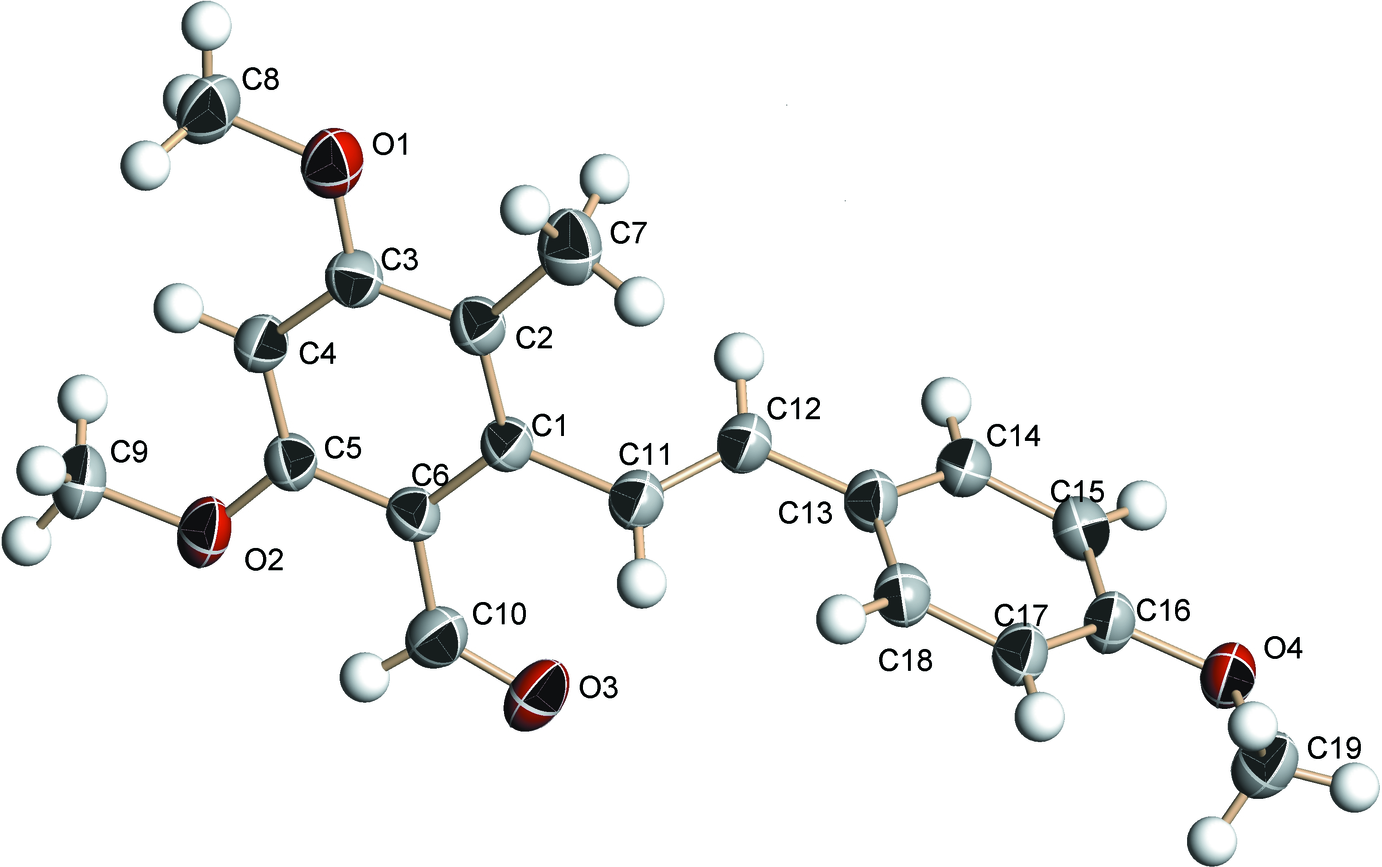

Supplement: Supplementary file 4 [file e-71-0o771-fig1.tif]

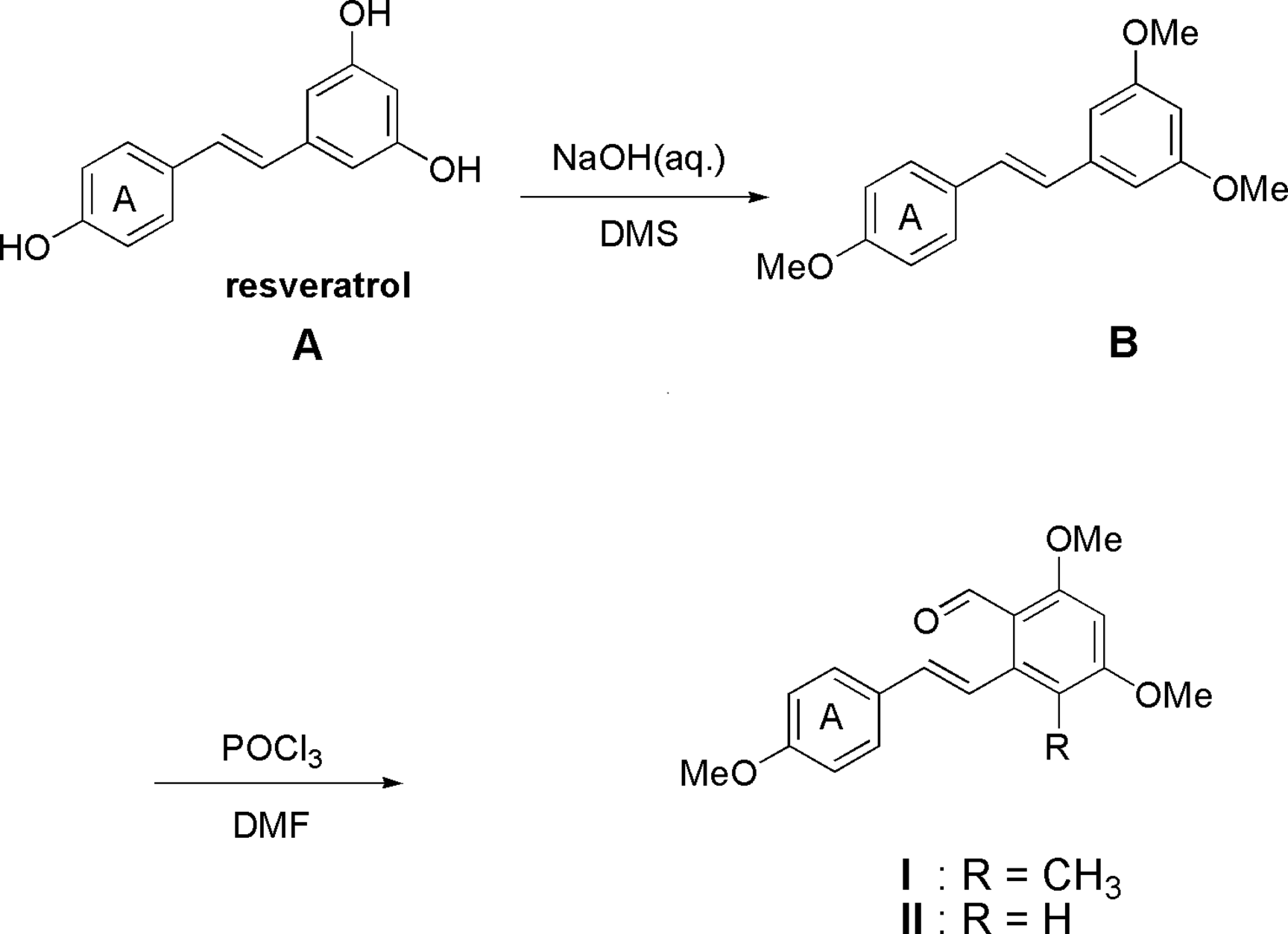

Supplement: Supplementary file 5 [file e-71-0o771-fig2.tif]
